# Severe Kidney Injury After a 110-km Trail Race

**DOI:** 10.7759/cureus.7814

**Published:** 2020-04-24

**Authors:** Volker Scheer

**Affiliations:** 1 Sports Medicine, Ultra Sports Science Foundation, Pierre-Benite, FRA

**Keywords:** : acute kidney injury, running-related injuries

## Abstract

We present a case of severe, acute kidney injury, rhabdomyolysis and dehydration in a 49-year-old, competitive trail runner, after a 110-km trail race in mountainous terrain. Six days after the event, he presented to the hospital with fatigue, weight gain and oedema. Biochemically the diagnosis of severe, acute kidney injury was made, with increased serum creatinine levels of 13.4 mg/dL (normal range 0.67-1.17 mg/dL). He remained hospitalised for two weeks, and improved with conservative measures, without the need for renal replacement therapy. Likely risk factors included ingestion of non-steroidal anti-inflammatory drugs prior to the event, dehydration and prolonged running in mountainous environment at moderate altitude. Renal function largely returned to baseline levels four months after initial presentation. This case highlights that severe kidney injury can occur, even days after ultra-running events, especially in the presence of associated risk factors. If repeated cases of acute kidney injury can trigger chronic kidney injury is currently unclear and further research in this area is warranted. In the meantime, efforts should be made to educate athletes, coaches and health care professionals about the dangers of acute kidney injury and associated risk factors.

## Introduction

Trail and off-road running events are popular, especially ultra-running distances in excess of marathon distance [1]. Medical problems can occur during these events but are mostly minor in nature, but serious and life-threatening conditions such as exercise-associated hyponatraemia, heat stroke, cardiovascular issues, rhabdomyolysis and acute kidney injury (AKI) can occur [2]. AKI is commonly seen after endurance running, with a prevalence of up to 80% during multistage ultra-running events, but is mostly self-limiting, and minor in nature, with renal function generally recovering fully within a few days [3,4]. Rhabdomyolysis is a condition defined by the disintegration of skeletal muscle after prolonged or strenuous exercise can lead to the release of muscle cell elements such as myoglobin and creatinine kinase (CK), which in turn can cause AKI [5]. Exertional heat stroke is also a cause of acute renal injury and can be observed in endurance runners [6,7]. Other risk factors for the development of AKI in running can include heat stress, dehydration, latent myopathy, ingestion of non-steroidal anti-inflammatory (NSAID) and infection [8,9].

## Case presentation

We present a case of severe AKI with rhabdomyolysis in a 49-year-old, experienced, competitive male trail runner (weight 66 kg, height 183 cm, body mass index 19.7 kg/m^2^, running 5-6 times/ week, training load 70-100 km/week) who presented six days after a 110-km trail race to the accident and emergency department. He participated in one of Europe’s toughest ultra-trail race, the Grossglockner Ultra-Trail, a 110-km trail race with 6.500 m positive climb and an average elevation higher than 2,000 m for at least half of the course, including technically difficult terrain and trails. He finished the race in a time of approximately 18 hours (overall winning time of 14:40:14 hr:min.sec). Prior to the event, he was free of injury, otherwise fit and healthy, non-smoker, with no other past medical history of note. Blood tests approximately six months before the event showed a normal renal function (creatinine 1 mg/dL, glomerular filtration rate [GFR] >90 mL/min/1.73 m^2^). Immediately prior to the event, he ingested 500 mg of naproxen and 20 mg of esomeprazole. Throughout the race, he consumed little oral fluids (approximately 4 litres of fluids in total, during approximately 18 hours of running). There was no urination throughout the race. Immediately after crossing the finish line, he started to re-hydrate and increased his fluid intake ad libitum and shortly thereafter had one episode of chocolate brown urination. He felt fine, with some post-race fatigue and subsequently returned home. The following day he noticed some mild generalised swelling with oliguria, despite regular food and fluid intake. After two days, urination returned to normal, but the generalised oedema increased over the following days with a combined weight gain of approximately 10 kg. Subsequently, he felt generally tired and fatigued, and at this stage he presented to the local accident and emergency department six days post-event. Biochemically the diagnosis of severe AKI with rhabdomyolysis was made. Conservative measures with intravenous and oral fluid therapy were instigated, and renal replacement therapy was contemplated. However, his kidney function gradually improved, as well as elevated potassium levels, with conservative measures; therefore renal replacement therapy was not required. Further investigations included abdominal radiographs (due to abdominal extension), ultrasounds of both kidneys, urine analysis (dip stick and microscopic) and serial laboratory blood tests, as well as cardiac assessment, including ECG. Radiological imaging and cardiological assessment were unremarkable, as well as blood gas analysis, without signs of metabolic acidosis. The patient remained hospitalised for two weeks, and on discharge his renal function and clinical symptoms had improved considerably. Subsequent regular blood test demonstrated a gradual return of his creatinine levels after approximately six months. Estimated laboratory GFR remained reduced, with no evidence of microproteinuria (albumin-to-creatinine ratio) (Table 1). At this point, he was allowed to return to gradual aerobic activity under close supervision of his sports physician.

**Table 1:** Values from biochemical markers at admission, discharge, four months and six months after presentation. Normal laboratory ranges are as follows: creatinine (0.67-1.17 mg/dL), urea (16.6-48.5, mg/dL), sodium (136-145 mmol/L), potassium (3.5-5.1 mmol/L), CRP (C- reactive protein; <0.5 mg/dL), myoglobin (28-72 g/L), CK (creatinine kinase; <190 U/L), LDH (lactate dehydrogenase; 135-225 U/L), GFR (glomerular filtration rate; >90 mL/min/1.73 m^2^).

Variables	Admission	Discharge	Four months	Six months
Creatinine (mg/dL)	13.4	2.9	1.2	1.17
Urea (mg/dL)	214	75	33	42
Sodium (mmol/L)	136	140	144	139
Potassium (mmol/L)	6.0	4.5	4.8	4.9
CRP (mg/dL)	1.1	0.1	<0.3	<0.3
Myoglobin (g/L)	264	80	<21	<21
CK (U/L)	1463	133	343	130
LDH (U/L)	2191	995	207	170
GFR (mL/min/1.73 m^2^)	7.0	47	67	70

## Discussion

The Acute Kidney Injury Network (AKIN) defines AKI as the sudden decrease of renal function, with an absolute increase of serum creatinine levels of at least 0.3 mg/dL or by a percentage increase of over 1.5× baseline value [10]. AKI in ultra-endurance runners is common with a prevalence of around 80%; however, most kidney injuries are mild and recover fully within a few days [3,11,12]. Our runner presented with an initial serum creatinine level of 13.4 mg/dL, an approximately 11-fold increase from baseline levels. This constitutes a severe case of AKI and falls within stage 3 of the AKIN classification, the most severe form of AKI [10].

Risk factors for AKI related to ultra-endurance running include acute dehydration, hypoxaemia, proinflammatory cytokine production, ingestion of NSAID, exertional heat stroke, rhabdomyolysis, viral or bacterial infection and a pre-existing condition [6,8,9,13].

We were able to identify several risk factors in our runner. Extrinsic factors, e.g., characteristics of the event itself, with a total distance of 110 km, important elevation changes and exercising at moderate altitude, can lead to an increased production of proinflammatory cytokines and mild hypoxaemia. The prolonged uphill and downhill running sections place an enormous demand on the musculature with an increase in destruction and release of muscle enzymes, such as CK. This was evidenced by persistently raised CK values (1,400 U/L, normal <190 U/L) even at first analyses six days post-event. Peak values are generally observed 24-48 hours post-event, with a gradual decline thereafter, so our values strongly suggest an important muscular damage post-race. The combination of these factors, in addition to the ingestion of NSAID, severe dehydration with anuria throughout the event, may all have contributed the severity of the kidney injury. The runner himself was unaware of the severity of the condition and only presented six days post-event to the hospital. It is speculative but likely that an earlier presentation to health care providers, with aggressive and early intervention, may have reduced the severity of the clinical picture. 

Although ultra-distance running does not impact short-term kidney function in the majority of runners, repeated AKI may trigger chronic kidney disease (CKD) [13]. If a severe case, as described here, may lead to accelerated progression to CKD is currently unknown. It may be worth screening athletes prior to ultra-endurance events and having baseline measurements. Additionally, there is currently no guidance for continued participation in ultra-endurance running events after such a severe kidney injury. A common-sense approach, with gradual return to preinjury running levels, under close medical supervision may be possible but needs to be evaluated on a case-by-case basis under expert supervision. It does however highlight the importance of providing awareness and education to athletes, coaches and health care professionals involved in ultra-endurance running, to avoid further cases like this and monitor and research long-term sequeales. 

## Conclusions

We present a case of severe AKI with rhabdomyolysis in a competitive master trail runner after a strenuous ultra-trail race. Mild-to-moderate AKI is common amongst ultra-endurance runners, but severe cases like this are extremely rare. The combination of various risk factors, including prior ingestion of NSAID, dehydration and competing at moderate altitude, may have led to this serious presentation. If repeated cases of AKI can trigger chronic kidney injury is currently unclear and further research in this area is warranted. In the meantime, efforts should be made to educate athletes, coaches and health care professionals about the dangers and signs of AKI and associated risk factors. 
